# The evolution of mutualism with modifiers

**DOI:** 10.1002/ece3.3180

**Published:** 2017-06-28

**Authors:** Christopher G. Quickfall, James A. R. Marshall

**Affiliations:** ^1^ Department of Computer Science University of Sheffield Sheffield UK; ^2^ Department of Animal and Plant Sciences University of Sheffield Sheffield UK

**Keywords:** altruism, assortment, donation, falsebeard, greenbeard, suppression

## Abstract

Mutualisms are widespread, yet their evolution has received less theoretical attention than within‐species social behaviors. Here, we extend previous models of unconditional pairwise interspecies social behavior, to consider selection for donation but also for donation‐suppressing modifiers. We present conditions under which modifiers that suppress costly donation receive either positive or negative selection; assortment only at the donation locus always leads to selection for donation suppression, as in within‐species greenbeard traits. However, genomewide assortment with modifier loci can lead to intermediate levels of donation, and these can differ in the two species even when payoffs from donation are additive and symmetric. When costly donation between species can evolve without being suppressed, we argue that it is most appropriately explained by indirect fitness benefits within the donating species, using partner species as vectors for altruism. Our work has implications for identifying both the stability and the ultimate beneficiaries of social behavior between species.

## 1 INTRODUCTION

Inclusive fitness theory explains the evolution of altruistic traits that help conspecifics at a lifetime personal fitness cost to their bearers (Hamilton, 1964a,b). When individuals within a species are genetically related, altruism can evolve because an actor can compensate for a lifetime cost to reproduction by helping recipients that are genetically similar. In this way, the recipients’ offspring can effectively replace the actor's offspring in the gene pool. However, social behavior can also occur between species, and there is growing empirical and theoretical interest in understanding the evolution of mutualism, which abounds in natural communities and is central to ecosystem form and function (Archetti et al., 2011; Bull & Rice, 1991; Fletcher & Doebeli, 2009; Fletcher & Zwick, 2006; Foster & Wenseleers, 2006; Frank, 1994, 1997; Gardner, West, & Wild, 2011; Herre, Knowlton, Mueller, & Rehner, 1999). Yet, the evolution of mutualism is arguably not as well understood as the evolution of within‐species social behavior.

To further develop mutualism theory, here we present a simple multilocus model of donation between species, where individuals of each species are given the opportunity to pay a cost to help a member of the other species. Others have considered similar systems from a community selection perspective (Goodnight, 1990); here, we analyze selection at only a single level, noting that the multilevel selection and inclusive fitness theory approaches make identical predictions on the direction of selection (Marshall, 2011, 2015).

Our model describes the evolution of costly donation, and suppression of donation. We are particularly interested in when suppression of such donation will and will not be stable, and compare scenarios whose main difference is whether association between species occurs at a subset of their genomes, or is genomewide. We analyze three cases; in the first, assortment at behavior‐generating loci brings heterospecific bearers of donation alleles together more frequently than expected under uniformly random assortment. However, it does not assort based on the other, potentially donation‐suppressing, locus; in other words, assortment is local rather than across the entire genome. This is analogous to an obligate greenbeard scenario within a single species; when the donation behavior is expressed, it is disproportionately aimed at other bearers of the donation allele. In the second case, genomewide assortment based on genotypes, a kind of interspecies relatedness, governs interactions; this is intended to correspond, as much as possible, with Hamilton's scenario for altruism in kin groups. In this case, assortment is no longer local. Finally, we alter the second case to consider a facultative greenbeard situation, in which donators only donate to other donators. We analyze all cases using quasi‐linkage equilibrium (QLE) (Kimura, 1965; Kirkpatrick, Johnson, & Barton, 2002) and stochastic simulation approaches. We present conditions under which modifiers that suppress individually costly donation receive either positive or negative selection. These conditions are similar to Hamilton's rule and are conceptually similar to analyses of selection acting on greenbeard traits within species. We also present conditions under which intermediate levels of donation in the two partner species are stable and further conditions under which these levels differ in the two species. We then consider how to characterize these interspecies social behaviors.

## 2 MATERIALS AND METHODS

In order to study the evolutionary stability of mutualism, we analyze models in which individuals of two species randomly pair up and have the possibility to help each other. We focus on a simple pairwise scenario because this reduces the potential for within‐species altruism that might be mistaken for between‐species altruism; for example, a honeybee collects nectar while pollinating a plant but uses it to raise siblings rather than offspring (Foster & Wenseleers, 2006; Frank, 1994; Wyatt, West, & Gardner, 2013). Nevertheless, we return to such examples in the discussion.

Previous models seeking to understand interspecies social behavior have focussed on unconditional behavior governed by single loci (Foster & Wenseleers, 2006; Frank, 1994; Wyatt et al., 2013). Our models consider the evolution of the loci that drive donation toward a second species, but also of modifiers acting to suppress that donation. We also consider, alternatively, modifiers that allow individuals to refuse social partners according to their genotype. The general model assumes biallelic loci for donation

(1) $$G=\left\{\begin{array}{cc} 1 & \text{if donor}\\ 0 & \text{otherwise} \end{array}\right.,$$

and suppressing donation

(2) $$X=\left\{\begin{array}{cc} 0 & \text{if donation‐suppressor}\\ 1 & \text{otherwise} \end{array}\right..$$

Then, personal fitness is defined as

(3) $$W=G^{\prime }X^{\prime }b-GXc,$$

where primes indicate partner's genotype (where the partner is from a different species) for the trait in question, and *b* and *c* are, respectively, the fitness benefits and costs of prosocial behavior received from and given to members of the other species. Note that individuals only donate if they possess the donation allele but lack the suppression allele, in which case they are referred to as donators; individuals that only hold the donation allele shall be referred to as bearers of the donation allele. Note our use of the term “donator” rather than “donor”; this is to convey that these individuals donate only under the right circumstances, which vary between the three considered cases. Importantly, we can use this model to ensure a lifetime personal fitness cost associated with donation, by specifying that each individual only interacts once during its lifetime and is randomly assigned the role of either potential donor or potential recipient (Quickfall, 2016).

Given the above model, donation is altruistic whenever *b *> *c* > 0; note that fecundity and personal fitness are equivalent in this case, as local competition is assumed not to take place (Hamilton, 1964a,b; Taylor, 1992; Wyatt et al., 2013). Our model makes use of the neighbor‐modulated fitness method of analyzing social evolution (Taylor & Frank, 1996), where “neighbors” are members of the partner species. We take the simplest case in which additive fitness costs, benefits, and assortment are the same from both species’ points of view; it is then sufficient to focus on only one of the species to ask whether personally costly donation in that species is ever stable.

We analyze this model using three methods, the Price equation (Appendices S1–3), QLE (Appendix S4: Section 4.2.1), and stochastic simulation (Appendix S4: Section 4.2.2). Analytic discussion of the Price approach is restricted to the appendix, as is some further explanation of the other two approaches. The QLE approach involves deterministically updating allele and genotype frequencies with each generation, according to recursive equations subject to the QLE approximation discussed by Kirkpatrick et al. (2002). This requires weak selection relative to recombination; the exact constraints are discussed in Appendix S4: Section 4.2.1. The stochastic approach is applied to the same scenarios as the QLE analysis, but introduces stochasticity and drops the QLE assumption of weak selection; this allows us to test the robustness of our results to stochasticity and strong selection. In both the QLE and simulation approaches, the two species are assumed to have constant and equal population size.

We first consider assortment on a single locus. This is similar to the thought‐experiment introduced by Fletcher and Doebeli (2009) in which a hypothetical experimenter manipulates interactions such that donating genotypes in each species are always paired (i.e., if *G* = 1, then *G*′ = 1, and vice versa); the main difference with this is the introduction of the suppression locus in our model which, crucially, is not subject to assortment in our first scenario. In the second scenario, genomewide assortment is introduced that matches the genotypes at donation and modifier loci between the two species and consequently also matches like phenotypes (Appendix S4: Section 4.1.1). This scenario is intended to correspond with, as much as possible, Hamilton's scenario for altruism in kin groups, and represents an extreme test case for the evolution of costly donation between species, in that we assume it provides the conditions most conducive for it.

In both scenarios, our assortment parameter, α, takes a value between 0 and 1 (representing uniformly‐at‐random pairing and maximal assortment, respectively), but the actual success of assortment is also dictated by the extent to which same‐genotype frequencies differ between the two populations. Thus, complete genotype or phenotype matching is not possible unless genotype frequencies are identical in the two populations (Gardner et al., 2011). When they are, this case becomes identical to the within‐species case, and the donation behavior evolves under the familiar condition of α > *c*/*b*, suggesting that the assortment parameter α resembles relatedness, just as it does in single‐population models (Grafen, 1979).

Finally, we consider an additional scenario in which donators can reject interactions with nondonators, thus fulfilling the conditions to be facultative greenbeards (Gardner & West, 2010). Genomewide assortment is once again applied here; the difference between this and the second scenario lies in rejection of interactions postassortment.

## 3 RESULTS

Our QLE and stochastic simulation (Appendix S4: Sections 4.2.1 and 4.2.2) approaches complement the Price equation analysis of the Appendix (S1–S3) by showing that when assortment occurs at a single behavior‐producing locus in each species, selection for donation suppression always occurs. Thus, under QLE and stochastic simulation analyses, donation to other species is unstable and transient. This can also be shown analytically by considering that fitness of nonsuppressors is strictly greater than that of suppressors, if there are some individuals with the donation allele (Appendix S4: Section 4.3.1).

**Figure 1:**
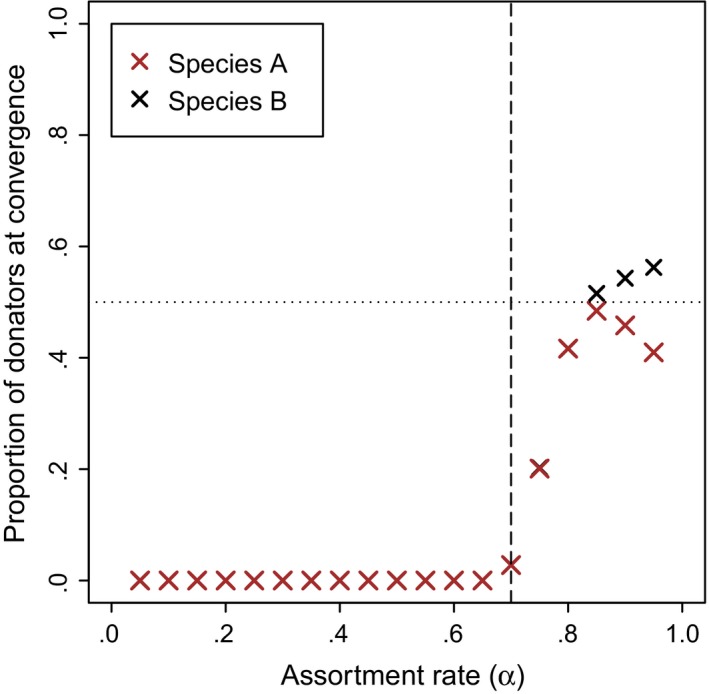
Stable proportions of donators in each species for intermediate whole‐genomic assortment rates α; model parameters are as described in the appendix. Stable donation evolves if α > *c/b* (vertical dashed line). Thus, something similar to Hamilton's rule then predicts the evolution of donation between species. However, if α sufficiently high such that donation frequency in both populations exceeds ½ (horizontal dashed line), more complex dynamics emerge and one species will donate less than the other on average (Appendix S4: Section 4.2.3.2)

Under our second scenario, in which assortment between species is genomewide rather than simply based on a single locus, the QLE analysis finds intermediate levels of donation in both species can occur (Fig. 1, Appendix S4: Section 4.3.2). When α > *c*/*b*, there are two types of stable population equilibrium; both are functions of α, *c* and *b*. The first exhibits equal frequencies of donation in the two populations, which increase linearly with α as it exceeds *c*/*b* (vertical dashed line in Fig. 1). As α increases beyond the point at which donation frequencies reach ½ (horizontal dashed line in Fig. 1), the frequencies in the two populations diverge. In both cases, the stable donation frequencies are dependent on α and *c*/*b*; analytic expressions are provided in Appendix S4: Section 4.3.2. Our results agree qualitatively with those of Foster and Wenseleers (2006), who identified a low *c*/*b* ratio and high between‐species fidelity as two of three primary factors which encourage mutualisms (the third, within‐species relatedness, is not systematically varied in our work). These results are insensitive to whether or not donators realize a net direct lifetime fitness benefit from interacting with like individuals (Appendix S4: Section 4.1) and are robust to finite population size and strong selection (Appendix S4: Section 4.3).

We now turn to our final scenario, in which donators can reject interactions with nondonators and thus act similarly to facultative greenbeards (Gardner & West, 2010). Assortment remains genomewide, as in our second scenario. In this case, donation reaches fixation if *b* > *c* (Quickfall, 2016). Note that the greenbeards account for the modifier, as it is implicit that greenbeards recognize their partner's allele on the “modifier” locus. Since greenbeards may typically be gene complexes (Gardner & West, 2010), this amounts to defining a new greenbeard trait that includes the modifier locus. Thus, these greenbeards are nonetheless vulnerable to additional modifiers that may arise at other loci. As with greenbeards, as discussed below, the only way that unlinked modifiers can receive negative selection is if silencing the donation aspect of the greenbeard necessarily leads to the loss of social benefits from other greenbeards.

## 4 DISCUSSION

**Figure 2:**
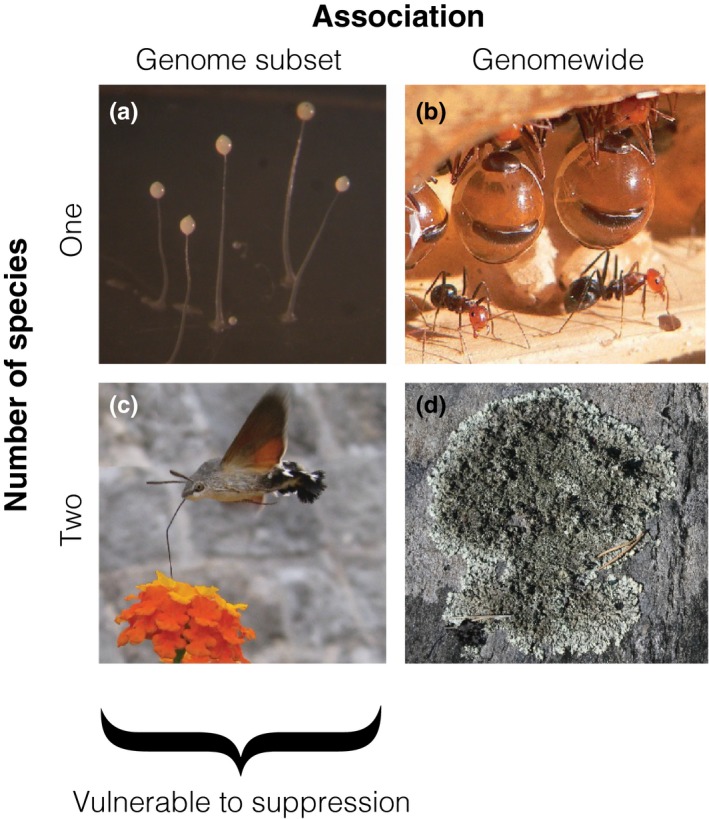
Conceptual framework for social behavior within and between species. Costly behaviors can experience positive selection either due to association between social alleles at a subset of the genome, or through genomewide association arising from population structure. Within single species, greenbeard theory shows that association between social alleles at a subset of the genome is vulnerable to suppression (illustrated by (a), greenbeard gene csA in *Dictyostelium discoideum* (Queller et al., 2003), as discussed in the main text); however, when relatedness is genomewide, social behavior is stable due to aligned inclusive fitness interests at all loci (illustrated by (b), sterile workers in honeypot ants). Our results show that this pattern is repeated in interactions between species; when association between species is at a subset of the genome, then social behavior is vulnerable to suppression (illustrated by (c), plant–pollinator associations, as discussed in the main text), but when association between species is genomewide, social behavior is stable and can be understood as due to inclusive fitness benefits with each species, using the partner species as a vector for altruism (illustrated by (d), the fungus‐algae/bacteria association in lichen, as discussed in the main text). Photographs (a,c,d) by Kevin Foster. Photograph (b) by Greg Hume, used under Creative Commons license (http://creativecommons.org/licenses/by/3.0/)

Our results on the evolution of between‐species costly behavior with modifiers show that when association between species occurs at a subset of the genome then suppression for donation at unassociated loci will always receive positive selection, whereas when association is genomewide then costly donation can be stable when something akin to Hamilton's rule is satisfied; as summarized in Fig. 2, this exactly matches the pattern for stability of costly social behaviors within species, as determined using inclusive fitness and greenbeard theory.

Greenbeards in general are vulnerable to suppression of the donation phenotype while preserving the marker that results in receipt of benefits. However, theoretically greenbeards are not vulnerable to suppression when the costly behavior cannot be dissociated from the marker that leads to receipt of benefits, and examples of this have been found in single species such as the csA gene in associations of amoebae (Fig. 2a) (Queller, Ponte, Bozzaro, & Strassmann, 2003) and the FLO1 gene in brewer's yeast (Smukalla et al., 2008), among others. What are the biological parallels to greenbeard associations and whole‐genome relatedness in two species associations? Greenbeard‐like association between species at a subset of their genomes may be possible. For example, in plant–pollinator associations (Fig. 2c), coevolution of flower and mouthpart designs may lead to stable association at portions of the species’ genomes associated with these traits, yet leave the rest of the genomes free to evolve modifiers to suppress the costly parts of these traits, namely investment in specialist morphology, while still reaping the benefits. In this example, there may be evidence of this on at least one side of the association, in the form of “nectar robbing” species benefiting from nectar without benefiting the plant by aiding in pollination (but see Maloof (2001) on whether this reduces plant fitness), and presumably thereby reducing the direct fitness cost of their behavior. However, the greenbeard metaphor may be rather stretched in such an example. Are interspecific stable greenbeards possible then? One candidate may be the cellular adhesion genes mentioned above in the context of single‐species associations, given the prevalence of multispecies microbial associations in the form of biofilms (Elias & Banin, 2012).

Genomewide relatedness in single species arises in family‐structured groups, which can provide the conditions necessary for the evolution of extreme altruism such as worker sterility in eusocial insects (Fig. 2b). Our results show that in two‐species associations, whole‐genome association also allows costly interspecific behaviors to be stable. Biological instances of such associations may seem hard to conceive of; we suggest that even if this is the case, theory has previously led the way to discovery of real biological phenomena, with greenbeard traits themselves being a case in point (Gardner & West, 2010). We further suggest that stable endosymbioses, particularly those in which endosymbionts are perfectly heritable, may provide the kind of interspecies association that corresponds to our model of costly interspecies donation.

Previous work has shown that costly interspecies donation can be analyzed as instances of within‐species altruism, but that a between species altruism interpretation is also possible (Wyatt et al., 2013). However, we argue that it is arbitrary to terminate the path of received benefits in the partner species, when it continues back to conspecific relatives of the focal actor. Thus, we favor treatment of costly between‐species donation as within‐species altruism, using the partner species as a vector (Queller, 2011). For example, lichens, which are symbiotic combinations of fungi and photosynthetic algae or cyanobacteria (Fig. 2d), meet this interpretation since the association is stable, and diffuse benefits from the partner species are necessarily received by conspecifics of any donating individual in either species. By doing so, we preserve a further analogue between greenbeard theory and mutualism theory. When possible modifiers favor suppression of greenbeards within and between species, this is because direct fitness is increased by doing so, with no corresponding reduction in indirect fitness. This is the case as social partners that no longer receive donation as a result are unrelated at the modifier locus on average (left hand column of Fig. 2). On the other hand, modifiers are disfavored under genomewide association within and between species because indirect fitness losses to genetic, within‐species, relatives from suppressing costly donation more than outweigh the direct fitness benefit from doing so (right hand column of Fig. 2). Of interest for future work would be to consider the impact of increasing within‐species relatedness on the selective pressure experienced by modifiers in mutualisms, given that within‐species relatedness in single‐locus models has been found to promote the evolution of mutualism (Foster & Wenseleers, 2006), and given that in models of greenbeards and modifiers in single species increased relatedness relaxes the selective pressure for suppression of greenbeard donation (Biernaskie, West, & Gardner, 2011).

We conclude by considering the potential biological relevance of our results. One result is that stable but different levels of donation in two partner species are possible even when fitness costs and benefits are additive and the same from both species’ point of view. In single‐species models, such mixed‐equilibria occur when fitness effects interact negatively nonadditively, and when relatedness is at an intermediate level (Marshall, 2015; Queller, 1984). Here, the effect is due to the addition of modifier loci, and the complicated evolutionary dynamics this introduces (Appendix S4: Section 4.3.2) (Quickfall, 2016)). It remains to be seen whether this is a mere mathematical curiosity, or whether aspects of it may be of relevance in understanding some real examples of mutualism. In addition, our conceptual links between the evolution of mutualism and greenbeard theory, and the conceptual richness of the latter (Biernaskie et al., 2011) of which we have explored only a subset here, should be of interest. These results and links arose from introducing modifiers into simple models of the evolution of mutualism, and we hope future research into this area will be motivated in consequence.

## CONFLICT OF INTEREST

None declared.
