# Supplementary material for: The evolution of mutualism with modifiers
Source: Ecol Evol. 2017 Jun 28;7(16):6114–8. doi: 10.1002/ece3.3180 (PMC5574765; doi:10.1002/ece3.3180)
Supplement: Supplementary file 1 [file ECE3-7-6114-s001.pdf]

Appendix S1: Conditions for the Selection of Donation and its Suppression (Sexual Model)

We wish to rewrite equations for the covariances required to predict evolutionary change in a social behaviour, derived using the Price equation in the usual way, as inequalities that predict when donation and suppression will receive positive selection. Thus we wish to rewrite:

(S1.1) $$\mathrm{Cov}(G,W) = \mathrm{Cov}(G,G'X')b - \mathrm{Cov}(G,GX)c,$$

(S1.2) and $$\mathrm{Cov}(X,W) = \mathrm{Cov}(X,G'X')b - \mathrm{Cov}(X,GX)c,$$

(S1.3) as $$\frac{\mathrm{Cov}(G,G')}{\mathrm{Var}(G)} > \frac{\mathrm{E}(X)c}{\mathrm{E}(X')b},$$

(S1.4) and $$\frac{\mathrm{Cov}(X,X')}{\mathrm{Var}(X)} < \frac{\mathrm{E}(G)c}{\mathrm{E}(G')b}.$$

Note that equations (S1.1) and (S1.2) capture the epistatic interactions between alleles for donation and for its suppression. That is, a donation allele only results in social behaviour in the absence of a suppressor allele (when $GX = 1$ or $G'X' = 1$), and a suppressor allele only changes behaviour when paired with a donation allele (since if $G = 0$ no behaviour is performed regardless of whether $X = 0$ or $X = 1$, whereas if $G = 1$ behaviour is suppressed only when $X = 0$). We first consider a model of sexual reproduction, and start by considering a modifier at an unlinked locus; note that we are not here assuming that all loci in the genome are unlinked but rather assuming that it is possible for a modifier mutation to arise that is

unlinked to the donation locus. For simplicity we make a further assumption of weak selection (which we relax in Appendix S3); the result of these two assumptions is that linkage disequilibrium between donation and suppression loci is zero (they are statistically independent, i.e. $\mathrm{Cov}(G, X) = 0$ and $\mathrm{Cov}(G', X') = 0$). Given that linkage disequilibrium between donation and suppression loci is zero, we can also see that modifier allele in self is statistically independent from donation alleles in self and partner and vice versa (i.e. $\mathrm{Cov}(GG', X) = 0$ and $\mathrm{Cov}(GG', X') = 0$), and hence that X and G, and X and G', are pairwise independent[1]. Finally, by the definition of covariance we know that if $\mathrm{Cov}(A, B) = 0$ then $\mathrm{E}(AB) = \mathrm{E}(A)\mathrm{E}(B)$; this enables us to move independent variables outside covariances that involve them. Putting all these facts together enables us to rewrite (S1.1) and (S1.2) as

$$\mathrm{Cov}(G, W) = \mathrm{Cov}(G, G')\mathrm{E}(X')b - \mathrm{Var}(G)\mathrm{E}(X)c, \tag{S1.5}$$

$$\text{and} \qquad \mathrm{Cov}(X, W) = \mathrm{Cov}(X, X')\mathrm{E}(X')b - \mathrm{Var}(X)\mathrm{E}(G)c \tag{S1.6}$$

respectively, while still taking account of the epistatic fitness interactions between donation and suppression alleles mentioned in the main text. We can then ask when (S1.5) and (S1.6) are positive and negative, respectively, and (assuming we are considering costly behaviour, i.e. $c > 0$) rearrange to

[1] This can be shown as follows: although G and G' and G and X can both be non-independent due to, respectively, relatedness and (potentially) linkage disequilibrium, we assume that G' and X are conditionally independent given G. So we have $P(GG'X) = P(G)P(G'|G)P(X|G)$. Given we know that G and X are independent this can be rewritten as $P(G)P(G'|G)P(X) = P(GG')P(X)$. Thus $P(GG'X) = P(GG')P(X)$ showing that GG' and X are independent. This indicates that X is independent of G (which we already assumed) and G' (which we wished to demonstrate).

give conditions (S1.3) and (S1.4). Conditions (S1.3) and (S1.4) have natural interpretations as Hamilton's rule with averaged costs and benefits, according to the probability of donation/suppression actually occurring within the population. Note that this averaging accounts for the epistatic interactions between the donation and suppression alleles; for example, there is a reduced cost of bearing a donation allele when it is very likely that the bearer also contains an allele for suppression of that donation i.e. $\mathrm{E}(X)$ is close to zero in the right-hand side numerator of inequality (S1.3). Note that the inequality in condition (S1.4) represents a condition for the increase in frequency of suppression (i.e. selfishness), hence it is reversed because of the way in which X represents suppression.
