# Supplementary material for: The evolution of mutualism with modifiers
Source: Ecol Evol. 2017 Jun 28;7(16):6114–8. doi: 10.1002/ece3.3180 (PMC5574765; doi:10.1002/ece3.3180)
Supplement: Supplementary file 2 [file ECE3-7-6114-s002.pdf]

Appendix S2: Conditions for Selection of Donation and its Suppression in the Interspecies Model of Fletcher and Doebeli (Sexual Model)

We wish to instantiate conditions (S1.3) and (S1.4), which predict respectively when donation and its suppression will receive positive selection, for the case where individuals are always only paired with other donators (i.e. when $G = 1$, $G' = 0$). This results in two new inequalities for that particular scenario. These are

$$\text{(S2.1)} \qquad \frac{\mathrm{E}(G)(1-\mathrm{E}(G'))}{\mathrm{E}(G)(1-\mathrm{E}(G))} > \frac{\mathrm{E}(X)c}{\mathrm{E}(X')b},$$

$$\text{(S2.2)} \qquad \text{and} \qquad 0 < \frac{\mathrm{E}(G)c}{\mathrm{E}(G')b}.$$

These are derived as follows. $\mathrm{Cov}(G, G')$ in condition (S1.3) equals $\mathrm{E}(GG') - \mathrm{E}(G)E(G')$ by definition of covariance. Since partner's genotype is 1 if and only if the focal individual's is also 1, the first expectation for the above scenario is equal to $\mathrm{E}(G)$, giving $\mathrm{Cov}(G, G') = \mathrm{E}(G)(1 - E(G'))$ in the numerator of the LHS of equation (S2.1). The denominator of the LHS is $\mathrm{Var}(G) = \mathrm{E}(G)(1 - \mathrm{E}(G))$ by definition of variance. For condition (S1.4) $\mathrm{Cov}(X, X') = 0$ (given the suppression loci are in linkage equilibrium with the donation loci in focal species and partner species, they are also statistically independent of each other, using a similar argument to that presented in Appendix S1 on the independence of donation locus and partner's suppression locus, and vice-versa); thus the LHS of inequality (S2.2) equals 0.

Furthermore, as noted in (Gardner et al., 2011), an implicit feature of this model imposed by the pairing scheme proposed, is that donator frequencies are the same in each population: $\mathrm{E}(G) = \mathrm{E}(G')$. Substituting this equality into conditions (S2.1) and (S2.2) and simplifying them gives

$$1 > \frac{\mathrm{E}(X)c}{\mathrm{E}(X')b}, \tag{S2.3}$$

and

$$0 < \frac{c}{b} \tag{S2.4}$$

This indicates that, while inter-specific donation will indeed be selected for according to Hamilton's rule (Gardner et al., 2011), as long as averaged benefits exceed averaged costs ($\mathrm{E}(X')b > \mathrm{E}(X)c$), suppression of donation will always be selected for at loci in linkage equilibrium. In fact, while zero linkage disequilibrium allows an intuitive analysis of selection for donation and its suppression, thereby highlighting the inclusive fitness logic of the process, it is not strictly necessary; an additional analysis shows equivalent results for asexual reproduction under strong selection (Appendix S3).

Finally, as noted in (Gardner et al., 2011), the inter-specific donation model proposed in (Fletcher & Doebeli, 2009) is akin to a greenbeard situation. Since greenbeard donation is vulnerable to suppression by modifiers, as it does not result in genome-wide-similarity between actors, it is not surprising that the scenario of (Fletcher & Doebeli, 2009) is also vulnerable to modifiers. In fact, the link with greenbeard donation and its suppres-

sion can be made more explicit by assuming a greenbeard trait in a single species (no change in the model is required, since the assumption that $E(G) = E(G')$ has already been made), and a single modifier locus in that species (so $E(X) = E(X')$). This results in a new selection condition for the greenbeard trait

$$1 > \frac{c}{b} \tag{S2.5}$$

indicating that donation is always favoured as long as benefits exceed costs (this agrees with the intuition that relatedness between interacting greenbeard donators is 1), while its suppression is also always favoured due to zero relatedness at loci in linkage equilibrium.

The general model could be applied to conditions where donation is governed by different loci in actor and neighbour within the same species e.g. (Fletcher & Doebeli, 2009), and similarly for modifiers for suppression in actor and neighbour. The model could also be applied to a single donation locus and a single modifier locus, governing interactions within a single species, in which case $E(G) = E(G')$ and $E(X) = E(X')$, so Hamilton's rule with costs and benefits in terms of direct payoffs from interactions is recovered from conditions (S1.5) and (S1.6). The crucial point to note is that the genetic association between actors at the donation locus and the modifier locus can be different, according to the means by which this association is generated. This ability for relatedness to differ across the genome is well known to be im-

portant for the evolution of social behaviours. For inter-specific interactions a key implication, as noted by Foster (2009), is that inter-specific donation that arises by matching at the donation locus will thus be vulnerable to donation-suppressing modifiers. This is because, in different species, genetic similarity at the donation locus will not guarantee genetic similarity at other loci.

One could attempt to rescue the 'altruism' scenario by Fletcher & Doebeli (2009) by introducing additional population structure, further associating individuals according to their modifier traits. However, new modifiers at unlinked loci could continually arise to suppress donation, requiring further population structuring, and so on ad infinitum. Thus inter-specific 'altruism' of the kind discussed by Fletcher & Doebeli (2009), while possible in principle and explainable by Hamilton's rule (Gardner et al., 2011), is not stable (Foster, 2009).

Bibliography

Fletcher, J.A. & Doebeli, M. 2009. A simple and general explanation for the evolution of altruism. *Proc. R. Soc. Lond. B Biol. Sci.* **276**: 13–9.

Foster, K.R. 2009. A defense of sociobiology. *Cold Spring Harb. Symp. Quant. Biol.* **74**: 403–18.

Gardner, A., West, S.A. & Wild, G. 2011. The genetical theory of kin selection. *J. Evol. Biol.* **24**: 1020–43.
