# Supplementary material for: The evolution of mutualism with modifiers
Source: Ecol Evol. 2017 Jun 28;7(16):6114–8. doi: 10.1002/ece3.3180 (PMC5574765; doi:10.1002/ece3.3180)
Supplement: Supplementary file 3 [file ECE3-7-6114-s003.pdf]

Appendix S3: Conditions for Selection of Donation and its Suppression in the Interspecies Model of Fletcher and Doebeli (Asexual Model)

The argument (Appendix S2) that suppression of inter-species-donation at unlinked loci will always receive positive selection is implicitly based on a sexual model, since physical non-linkage between donation and suppression loci is assumed. The analysis also assumes weak selection, since zero linkage disequilibrium between donation and suppression loci is assumed. We now consider a model of inter-species donation involving an asexual focal species, that relaxes both of these assumptions. Personal fitness is as defined in equation (3) in the main text, which we reproduce here for convenience as

$$W = G'X'b - GXc \tag{S3.1}$$

We then wish to consider when suppression of donation will receive positive selection, i.e. when equation (S1.2) is negative. That is, we wish to solve for when

$$\mathrm{Cov}(X, W) = \mathrm{Cov}(X, G'X')b - \mathrm{Cov}(X, GX)c \tag{S3.2}$$

is negative. This time, however, we cannot average the effects of epistatic interactions due to independence of loci, as in Appendix S1, but rather solve

(S3.3)
$$\frac{\mathrm{Cov}(X, G'X')}{\mathrm{Cov}(X, GX)} < \frac{c}{b},$$

by taking account of all possible genotypic combinations of donation and modifier alleles; there are four such possible genotypes to consider. We keep track of the proportions of the population that have the $G = X = 1$ genotype (whose frequency is denoted g), the $G = 1, X = 0$ genotype (frequency f), and the $G = 0, X = 1$ genotype (frequency h). The remaining proportion $1 - g - f - h$ of the population thus have genotype $G = X = 0$. Given the pairing scheme proposed by Fletcher & Doebeli (2009), we have that $G' = 1$ if and only if $G = 1$, hence relatedness at the donation locus is 1; then the only uncertainty is whether actor and partner are bearers of the donation suppression allele ($X = 0$) or not ($X = 1$). The probabilities of the different pairings are given in table 1 below. By summing over the relevant rows of table 1 we calculate $\mathrm{E}(XG'X') = g^2$ and $\mathrm{E}(X)\mathrm{E}(G'X') = (g + h)g$, so we have $\mathrm{Cov}(X, G'X') = -gh$.

Similarly, since $\mathrm{E}(GX^2) = g$ and $\mathrm{E}(X)\mathrm{E}(GX) = (g + h)g$ we have $\mathrm{Cov}(X, GX) = g(1 - g^2 - h)$. Thus inequality (S3.3) can be rewritten as

(S3.4)
$$-\frac{h}{1 - g^2 - h} < \frac{c}{b}$$

G	G'	X	X'	Probability
0	0	0	0	$(1-g-f-h)^2$
0	0	0	1	$h(1-g-f-h)$
0	0	1	0	$h(1-g-f-h)$
0	0	1	1	h^2
0	1	0	0	0
0	1	0	1	0
0	1	1	0	0
0	1	1	1	0
1	0	0	0	0
1	0	0	1	0
1	0	1	0	0
1	0	1	1	0
1	1	0	0	f^2
1	1	0	1	fg
1	1	1	0	fg
1	1	1	1	g^2

Table S3.1: Probabilities of different genotype pairings given the experimental scheme proposed by Fletcher & Doebeli (2009).

which, since the LHS is always negative and the RHS always positive, shows that suppression of donation in the asexual model always receives positive selection, just as in the sexual model with traits in linkage equilibrium.

Using Table S3.1 we can also calculate $\mathrm{Cov}(X, X') = \mathrm{E}(XX') - \mathrm{E}(X)\mathrm{E}(X') = g^2 + h^2 - (g + h)^2 = -2hg$, indicating that relatedness at the modifier locus is always negative (since to get the relatedness we divide $\mathrm{Cov}(X, X')$ by $\mathrm{Var}(X)$, which is always positive by definition). However, if $g = 0$ or $h = 0$, i.e. modifiers are always found with donators or always found with non-donators, then relatedness at the modifier locus is zero.

We can also calculate linkage disequilibrium as $\mathrm{Cov}(G, X) = \mathrm{E}(GX) - \mathrm{E}(G)\mathrm{E}(X) = g-(f+g)(g+h)$. This can be negative or positive, and will only be zero for a limited number of genotype frequencies, thus demonstrating that the assumption of zero linkage disequilibrium is not needed for suppression of donation always to receive positive selection.

Bibliography

Fletcher, J.A. & Doebeli, M. 2009. A simple and general explanation for the evolution of altruism. *Proc. R. Soc. Lond. B Biol. Sci.* **276**: 13–9.
