# Supplementary material for: The evolution of mutualism with modifiers
Source: Ecol Evol. 2017 Jun 28;7(16):6114–8. doi: 10.1002/ece3.3180 (PMC5574765; doi:10.1002/ece3.3180)
Supplement: Supplementary file 4 [file ECE3-7-6114-s004.pdf]

Appendix S4: Details of the Between-Species Donation/Suppression Model

S4.1 Model Specifications and Formulations

We consider a two-locus, biallelic model with two haploid populations of equal size. The two populations represent two different species; all interactions are interspecific, and individuals act as both potential donators and potential recipients (though the following results hold even if individuals are uniformly at random assigned a single role in their lifetime (Quickfall, 2016a)). The first locus represents donation and non-donation, and the second locus represents suppression and non-suppression. Individuals unconditionally donate, and are labelled donators, if and only if they possess the donation and non-suppression alleles. All other individuals do not donate. The cost associated with donation behaviour is c, and the benefit conferred to the social partner is b; we constrain the two so that $0 < c < b$ and $c < w$, where w is the baseline fitness. We set w to be 1; we lose no generality, since c and b are both scaled to w.

We explore various modifications of this basic model. In model (a), assortment (represented by our parameter α) is applied to only the donation locus, whereas in model (b), it is applied to the whole genome. In model (c), donators no longer donate unconditionally; instead they only donate if their social partner is also a donator.

We discuss the details relevant to the construction of model (b) only, for simplicity, since many of the details are relevant to models (a) (which involves only an alteration to the β function) and (c) (which involves only an alteration to the fitness functions). First, however, we define some notation; the subscript i refers to the population index of the focal individual (or population), and the subscript $-i$ refers to the index of the other population. We let the frequency of the donation allele in population i be $p_{1,i}$, and the frequency of the non-suppression allele in population i be $p_{2,i}$. The frequencies of non-donation and suppression alleles are $q_{1,i} := 1 - p_{1,i}$ and $q_{2,i} := 1 - p_{2,i}$ respectively. The allele at the donation locus for an individual is represented by a 1 if the individual bears the donation allele, and 0 if they have the non-donation allele. Similarly, the allele at the suppression locus for an individual is represented by a 1 if the individual has the non-suppression allele, or a 0 if the individual has the suppression allele. Thus, the set of genotypes is G={(0,0),(0,1),(1,0),(1,1)}, where donators are (1,1) individuals.

S4.1.1 The Assortment Parameter

Next, we let $P_i(u, v)$ be the probability that an individual with genotype u is matched up with a v-genotype individual, which is necessarily a member of population $-i$. Let $f_i(u)$ be the frequency of the genotype u in the ith population.

We construct a function which gives the probability of a focal individual

with genotype $u \in G$ from population i being matched up with a social partner with genotype $v \in G$ from population $-i$. This is labelled P, and must be constructed in such a way that it applies generally to each model (note that it could easily be adapted to consider only alleles in the single-locus assortment case, though we do not here (Quickfall, 2016a)), and satisfies the following two conditions:

$$\text{(S4.1)} \qquad \sum_{v \in G} P_i(u, v) = 1 \quad \forall u \in G$$

$$\text{(S4.2)} \qquad \sum_{u \in G} f_i(u) P_i(u, v) = f_{-i}(y) \quad \forall v \in G$$

The condition (S4.1) states that the sum of the matching probabilities for a genotype u is 1. The second condition, (S4.2), states that the sum of the frequencies of each genotype multiplied by the probability that they are matched with a given genotype, is the frequency of the given genotype in the other population. This must be true, otherwise the pairings for each population would not correspond to one another.

We construct P in several stages. Firstly, we create a 'bias' function, which measures the frequency of pairings between individuals with genotype u from population i with individuals of genotype v in the other population; this is denoted $\beta_i(u, v)$. The exact form of the assortment parameter varies between models; we only present details of the genome-wide assortment model (b) here. Assortment is non-negative, so $\beta_i(u, v) \geq 0$ for all

$i \in \{1, 2\}$ and $u, v \in G$. Alike individuals are paired up according to the bias function and α, before the remaining individuals are paired up uniformly at random.

Note that the maximum frequency of paired individuals of genotype t is $\min(f_1(t), f_2(t))$. This is also the maximum of the bias function; the minimum of the bias function is 0. We introduce the assortment parameter as $\alpha \in (0, 1)$. It takes a value in this range because we weight the amount of bias by α. Thus, we have found our bias function for model (b):

$$\beta_i(u, v) = \alpha I_{\{u=v\}} \min(f_i(u), f_{-i}(u)) \tag{S4.3}$$

Under model (b) only individuals of the same genotype are paired up by assortment; thus, we include an indicator function, $I_{\{u=v\}}$, to specify that assortment only take place between individuals of the same genotype. Next, we find that the unmatched genotype frequencies once the bias has been taken into account are:

$$g_i(u) = f_i(u) - \sum_{v \in G} \beta_i(u, v). \tag{S4.4}$$

This is a general g function; it allows for individuals with different genotypes to be matched, as indeed they are under single-locus assortment (model (a)).

Necessarily, we know that $\sum_{u\in G} g_1(u) = \sum_{v\in G} g_2(v)$, as the number of individuals left unmatched by association from both populations must be the same, since we have assumed that populations are of equal size. We only have a proportion $\sum_{u\in G} g_1(u)$ of individuals left to match up, so the frequency of individuals matched after association is $g_i(u)g_{-i}(v)/\sum_{u\in G} g_1(u)$ (for genotype u in population i matched with genotype v in the other population). Thus, we reach the final expression for P:

$$P_i(u,v) = \frac{\beta_i(u,v)}{f_i(u)} + \frac{g_i(u)g_{-i}(v)}{f_i(u)\sum_{t\in G} g_1(t)} \tag{S4.5}$$

Note that the first component of the RHS, which denotes the probability of being matched by the assortment mechanism, is only non-zero if the two individuals are paired up by assortment. The second component relates to matching after assortment. It is straightforward to prove that (S4.5) satisfies conditions (S4.1) and (S4.2).

Thus, we find that the average fitnesses of individuals with genotype u (denoted $W_i(u)$) in the ith population are as follows:

(S4.6a) $$W_i(0,0) = w + P_i((0,0),(1,1))b$$

(S4.6b) $$W_i(0,1) = w + P_i((0,1),(1,1))b$$

(S4.6c) $$W_i(1,0) = w + P_i((1,0),(1,1))b$$

(S4.6d) $$W_i(1,1) = w + P_i((1,1),(1,1))b - c$$

We are interested in whether donators will receive positive or negative selection. We can express this by using alternative notation based on phenotype, with donators represented by a 1, and non-donators represented by a 0. We then denote the frequency of pairings between individuals of type x in the focal population to individuals of type y in the other population as $Q(x, y)$. Sums over a type are represented by an asterisk - e.g. the frequency of pairings where the focal individual is a donator is equal to $Q(1, *)$. Thus, by comparing the average fitness of donators against the average fitness over all non-donators, we reach the following condition for positive selection on donators in the population of interest (Quickfall, 2016a):

(S4.7) $$\frac{Q(1,1)Q(0,*) - Q(0,1)Q(1,*)}{Q(0,*)Q(1,*)} > \frac{c}{b}$$

Note that this expression is only valid when $Q(0, *)$ and $Q(1, *)$ are non-zero. We shall return to (S4.7) later on while discussing results.

S4.2 The Two Modelling Approaches

We consider two methods of analysing this model, both of which utilise the assortment parameter α. One is a quasi-linkage equilibrium approach, and the other approach uses an individual-based stochastic simulation. Note that code relating to each method for each model, in addition to three models only briefly mentioned this appendix, is available online online (Quickfall, 2016b).

S4.2.1 The QLE Approximation and Deterministic Model

The crucial assumption we make in the following analysis is that the two populations are in quasi-linkage equilibrium (QLE; sometimes referred to as quasi-gametic equilibrium or QGE in the literature); a state originally investigated by Kimura (1965). This states that under certain assumptions, linkage disequilibrium (D) reduces to a low value which depends on allele frequencies and selection, but not previous values of D (Rice, 2004). Firstly, we must have that $a \ll 1$, where a is the greatest of the selection coefficients as defined by Kirkpatrick et al. (2002). Secondly, the linkage disequilibrium within population i, D_i, must always be of the order a. We only consider one value of linkage disequilibrium for each population since the only set of loci across which it can be defined is $\{1, 2\}$.

S4.2.1.1 Requirements of QLE

The first requirement of QLE is that the selection coefficients as defined by Kirkpatrick et al. (2002) are far smaller than 1. These are defined according to the following equation (adapted from equation (7) in Kirkpatrick et al. (2002)):

$$W_i(z) = \bar{W}_i(1 + \sum_U a_{U,i}(Y_{U,i,z} - D_{U,i})) \tag{S4.8}$$

Here, $\bar{W}_i$ is the mean fitness for population i, $U := \{1, 2, \{1, 2\}\}$, $Y_{U,i,z}$ is the deviation of the allele value from the mean allele value on locus U in population i (e.g. if $U = 1$, $x = 0$, and $i = 1$, then $Y_{U,i,z} = q_{1,1} = 1 - p_{1,1}$), and $Y_{\{1,2\},i,z} := Y_{1,i,z}Y_{2,i,z}$. We also have $D_{1,i} = D_{2,i} = 0$, and $D_i := D_{\{1,2\},i}$. Here, D_i is the linkage disequilibrium pertaining to the ith population; there is only one value associated with each population, as there are only two loci. Linkage disequilibrium can be thought of as the covariance between the donation and non-suppression alleles within a population (Gardner et al., 2007). The three selection coefficients for each population i are $a_{1,i}$, $a_{2,i}$, and $a_{\{1,2\},i}$. They correspond to a fitness adjustment to an individual using information on the locus (or loci) specified in the subscript of the a's. The adjustment is weighted by the mean population fitness and by the deviation of their allele value from the mean allele value; for example, a donator will have fitness given by the following expression:

$$W_i(1,1) = \bar{W}_i(1 + a_1(1-p_1) + a_2(1-p_2) + a_{\{1,2\}}(1-p_1)(1-p_2)) \tag{S4.9}$$

The first term in the parentheses corresponds to the average fitness, the second corresponds to the adjustment for possessing the donation allele, the third corresponds to the adjustment for possessing the non-suppression allele, and the final term corresponds to the adjustment from possessing both alleles; these are all weighted by the mean population fitness. Note that the adjustments can be either positive or negative. Once again, the equations change according to the model considered; here we present only genome-wide assortment, model (b). Similar analyses have been undertaken for models (a) and (c) (Quickfall, 2016a), however are not presented here for brevity.

We use equations (A18-22) to obtain selection coefficients for the ith population:

$$a_{1,i} = \frac{\tilde{b}(P_i((1,0),(1,1)) - P_i((0,0),(1,1)))}{1 + \tilde{b}f_{-i}(1,1) - \tilde{c}f_i(1,1)} + p_{2,i}a_{\{1,2\},i} \tag{S4.10a}$$

$$a_{2,i} = \frac{\tilde{b}(P_i((0,1),(1,1)) - P_i((0,0),(1,1)))}{1 + \tilde{b}f_{-i}(1,1) - \tilde{c}f_i(1,1)} + p_{1,i}a_{\{1,2\},i} \tag{S4.10b}$$

$$a_{\{1,2\},i} = \frac{\tilde{b}\big(P_i((1,1),(1,1)) + P_i((0,0),(1,1))\big)}{1 + \tilde{b}f_{-i}(1,1) - \tilde{c}f_i(1,1)} + \frac{\tilde{b}\big(-P_i((1,0),(1,1)) - P_i((0,1),(1,1))\big) - \tilde{c}}{1 + \tilde{b}f_{-i}(1,1) - \tilde{c}f_i(1,1)} \tag{S4.10c}$$

Here, we set $\tilde{c} = c/w$ and $\tilde{b} = b/w$ for simplicity. As mentioned earlier, the

first requirement for QLE is that the maximum of the selection coefficients is much less than 1. Thus, we let a denote $\max(|a_{1,1}|, |a_{2,1}|, |a_{\{1,2\},1}|))$. We note that $\max|a_{1,i}| < \max|a_{\{1,2\},i}|$ and $\max|a_{2,i}| < \max|a_{\{1,2\},i}|$. By noting that each P_i expression falls between 0 and 1, we find:

$$|a_{\{1,2\},i}| \leq \frac{2\tilde{b} - \tilde{c}}{1 - \tilde{c}} \tag{S4.11}$$

Thus, the first assumption of QLE, that $a \ll 1$, is satisfied if $\tilde{b} \ll 1/2$. This corresponds to weak selection, since both b and c are small relative to the baseline fitness, w, which we have already taken to be 1. Therefore, satisfying the conditions for QLE equates to a constrained range of parameter values that ensure weak selection.

We now turn to the second requirement for QLE; that is, that all values of D_i are of order a. Since $a < 2\tilde{b} - \tilde{c}/1 - \tilde{c}$, and necessarily, $D_i \leq 1/4$ (since $D_i := f_i(1,1) - p_{1,i}p_{2,i}$; this is maximised when $p_{1,i} = p_{2,i} = 0.5$, and $f_i(1,1) = 0$), we can see that:

$$D_i < \frac{4a(1 - \tilde{c})}{2\tilde{b} - \tilde{c}}$$

Thus, the conditions for QLE are met. We can find conditions for positive selection on the donation and non-suppression alleles, by using equations (S4.9) and (S4.10a-c). Both conditions involve the donation or non-

suppression alleles being selected for if some function of genotype frequencies and α is greater than c/b. In every single case, we can find a similar condition (Quickfall, 2016a). For model (b), the condition is mathematically intractable, and consequently not displayed. However, we can find that donators reach fixation in model (b) if $\alpha > c/b$, initial allele and genotype frequencies are the same, and there is no stochasticity (an entirely unrealistic condition); in model (c), PDs always evolve to fixation.

S4.2.1.2 Specifications of the Deterministic Model

We start with some initial allele frequencies, $p_{1,1}$, $p_{1,2}$, $p_{2,1}$, and $p_{2,2}$, which define the other initial frequencies $q_{1,1} = 1 - p_{1,1}$, $q_{1,2} = 1 - p_{1,2}$, $q_{2,1} = 1 - p_{2,1}$ and $q_{2,2} = 1 - p_{2,2}$. We are interested in whether PDs can invade a population when donation and suppression alleles are initially rare, so we choose $p_{1,1} = p_{1,2} = 0.1, p_{2,1} = p_{2,2} = 0.9$. We have tested more extreme initial frequencies (e.g. 0.01 initial donation and suppression), which always resulted in the same endpoints. However, we present results from the more moderate scenarios as they are more easily interpreted visually, and are computed more quickly. The parameters of the system that we shall choose in every test are the recombination rate, x, the baseline fitness w, and the benefits and costs associated with donation behaviour, respectively, b and c. We vary these initial conditions and parameters to investigate the system, though many results are not displayed for brevity (Quickfall, 2016a). The initial values of $a_{\{1,2\},i}, a_{1,i}$ and $a_{2,i}$ are found using these initial conditions and parameters and equations (S4.10a-c); in each successive iteration, these selection coefficients are updated.

We then find the change in the allele frequencies between two successive generations based on selection, using the following equations (derived using equation (10) in Kirkpatrick et al. (2002)):

(S4.12a) $$\Delta p_{1,i} = a_{1,i}p_{1,i}q_{1,i} + a_{2,i}D_i + a_{12,i}(1 - 2p_{2,i})D_i$$

(S4.12b) $$\Delta p_{2,i} = a_{2,i}p_{2,i}q_{2,i} + a_{1,i}D_i + a_{12,i}(1 - 2p_{1,i})D_i$$

Note the specific order in which equations are implemented: selection coefficients are defined by the allele frequencies and linkage disequilibrium, so need to be defined prior to the change in allele frequencies, which is calculated using the selection coefficients. Finally, the new value of D needs to be calculated at the end of every generation; this takes into account the effects of selection in the previous generation, and the new allele frequencies. The following expression finds our new values of D_i, derived using equation (19) in Kirkpatrick et al. (2002):

(S4.13) $$D_i = \frac{a_{12,i}p_{1,i}p_{2,i}q_{1,i}q_{2,i}}{x_i}$$

The process is terminated once the sum of the absolute changes in genotype frequencies between successive generations falls below a certain threshold, or alternatively after a specified number of generations in order to aid visual comparison of different results.

S4.2.2 The Stochastic Model

We consider a stochastic model for two reasons; firstly, to drop the assumptions of QLE, which entail determinism, infinite populations, and weak selection, and secondly, to either confirm the results of the QLE analysis, or understand what causes any differences. Thus, we are able to extend parameter values beyond those previously tested.

We start with a randomly generated finite population, as opposed to the deterministic, infinite population that we were working with previously. Pairings, fitness, breeding and mutations are simulated for each member of the two populations in each iteration. We use stochastic universal sampling (SUS) (Baker, 1987) when selecting individuals to breed, both to ensure that there is zero bias in expected amount of reproduction in comparison to sampling probability, and to reduce the amount of random number generation required, hence reducing computational complexity.

S4.3 Results

S4.3.1 Model (a): Single-locus Assortment

This model considers two populations of individuals who are paired according to assortment solely on the donation locus, and act as both potential donators and potential recipients. Analytically, we find, through comparison

of selection coefficients as detailed by Quickfall (2016a), that bearers of the suppression allele will always have equal or greater fitness than bearers of the non-suppression allele in the absence of assortment on the suppression locus. This is because assortment only applies to the donation locus - the suppression/non-suppression allele is 'invisible' to social partners. Individuals with the donation allele lose c fitness if they do not possess the suppression allele; individuals with the non-donation allele neither gain nor lose fitness on the basis of their allele on the suppression/non-suppression locus. Thus, so long as there are some donators, suppression will receive positive selection. Through testing we observe that recombination has a negligible effect on genotype frequencies (Quickfall, 2016a). We can also show, analytically, that the smaller the c/b ratio, the stronger the selection received by the donation and suppression alleles (Quickfall, 2016a). Both of these analytic results are borne out by the results of our QLE analysis - in particular, this confirms that the donation behaviour is transient. Further, these results are confirmed by stochastic simulation.; together, our analyses confirm that our results are robust to strong selection and finite population sizes.

S4.3.2 Model (b): Genome-wide Assortment

This model considers two populations of individuals who are paired according to genome-wide assortment, and act as both potential donators and potential recipients. The main conclusion from the analytic approach to looking at this model is that the c/b threshold is crucial to determining whether the donation and non-suppression alleles receive positive selection; some func-

tion of α, genotype and allele frequencies must be above this threshold for this to be the case. It can be shown analytically that changing the baseline fitness makes no difference to the outcome of the model, and we also confirm through simulation that the amount of recombination has almost no effect (Quickfall, 2016a). Thus, the importance of c/b, α, and the allele and genotype frequencies in determining whether the donation and non-suppression alleles receive positive selection is underlined. We can also show, analytically, that this model is equivalent to the alternative of allowing each individual to be either potential recipient or potential donator only once in their lifetimes (Quickfall, 2016a).

Intuitively, one would expect that a maximal amount of assortment will aid the evolution of donators, as when assortment in maximised, donators associate with one another as much as possible. However, donators do not go to fixation under maximal assortment; instead, donator frequencies are cyclic, since perfect assortment can leave individuals of certain genotypes with high probabilities of being paired with donators, due to frequencies of unassorted individuals. Thus, we consider only intermediate assortment. Under intermediate assortment, stable equilibria arise in which the two species have intermediate levels of donators if $\alpha > c/b$. Fig. 1 (main paper) shows the stable equilibria of donator frequencies in each species for intermediate levels of assortment, when $c/b = 1/2$. In fact, we can find the exact equilibria analytically - when $c/b = 1/2$ and $1/2 < \alpha \leq 2/3$, then $f_1(1,1) = f_2(1,1) = \frac{\alpha - c/b}{\alpha(1-c/b)}$. Similarly, if $\alpha \geq 2/3$, then equilibria exist at $f_1(1,1) = \frac{c/b}{\alpha(1+c/b)}, f_2(1,1) = \frac{\alpha(1+c/b)-c/b}{\alpha(1+c/b)}$ (Quickfall, 2016a).

The following verbal reasoning illustrates why it is possible for equilibria in which donator frequencies are unequal are possible to arise. Assume that two populations have different frequencies of donators. Donators in the population with more donators have smaller fitness than those in the population with fewer donators, as they have a smaller chance of being paired with donators. This corresponds to the $Q(1,1)Q(0,*)$ part of equation (S4.7) being smaller for the population with more donators. On the other hand, non-donators in the population with more donators will also have a smaller chance of being paired with donators, in comparison to non-donators from the other population, for the same reasons. This corresponds to the $Q(1,*)Q(0,1)$ component of equation (S4.7) also being smaller for the population with more donators. Thus, it is conceivable that donators and non-donators can have the same fitness within populations, even though individuals in the population with fewer donators will have greater fitness on average. The equilibria depicted in Fig. 1 (main paper) simply depict the exact points at which this is the case.

S4.3.3 Model (c): Genome-wide Assortment with Rejection of Interactions

This model is simply model (b), with the alteration that donators can reject interactions with non-donators. We find analytically that donators reach fixation whenever $b > c$, and confirm this through both QLE and stochastic simulation (Quickfall, 2016a).

However, unlike in the genome-wide assortment scenario described by model (b), we can note that these donators are facultative green-beards, since they possess the donation trait, recognise it in others, and only give preferential treatment towards individuals with this trait. The possibility of rejection ensures that PDs never lose any fitness, since we assume that $b > c$. As a result, this behaviour is mutually beneficial. Further discussion is located in the main text.

S4.4 Discussion

We have analysed three models of donation and suppression, finding that under single-locus assortment, the donation behaviour is transient, whereas under genome-wide assortment, a stable intermediate frequency of donators can evolve. We have not considered the possibility that a further modifier suppression locus might arise. If this emerged and there was no assortment on this locus, then using the argument for why donators were transient in model (a), we can see that it would not evolve while assortment does not apply to this locus. Finally, we considered the introduction of partner rejections; this ensures that donators reach fixation, but are facultative greenbeards, and thus only receive positive selection due to conditional donation.

Bibliography

Baker, J.E. 1987. Reducing bias and inefficiency in the selection algorithm. In: *Proc. Second Internat. Conf. on Genetic Algorithms*, pp. 14–21.

Gardner, A., West, S.A. & Barton, N.H. 2007. The relation between multilocus population genetics and social evolution theory. *Am. Nat.* **169**: 207–26.

Kimura, M. 1965. Attainment of quasi linkage equilibrium when gene frequencies are changing by natural selection. *Genetics* **52**: 875–90.

Kirkpatrick, M., Johnson, T. & Barton, N. 2002. General models of multilocus evolution. *Genetics* **161**: 1727–50.

Quickfall, C.G. 2016a. *Models of the Major Evolutionary Transitions (under revision)*. Ph.D. thesis, University of Sheffield.

Quickfall, C.G. 2016b. The evolution of mutualism with modifiers: Code and selected Workspaces. Retrieved from osf.io/rm6kh.

Rice, S.H. 2004. *Evolutionary Theory: Mathematical and Conceptual Foundations*. Sinauer Associates.
